# Integrins are not essential for entry of coxsackievirus A9 into SW480 human colon adenocarcinoma cells

**DOI:** 10.1186/s12985-016-0619-y

**Published:** 2016-10-18

**Authors:** Outi Heikkilä, Pirjo Merilahti, Marika Hakanen, Eveliina Karelehto, Jonna Alanko, Maria Sukki, Saija Kiljunen, Petri Susi

**Affiliations:** 1 Department of Virology, University of Turku, Turku, Finland; 2 Department of Medical Microbiology, Laboratory of Clinical Virology, Academic Medical Center, Amsterdam, The Netherlands; 3 Turku Centre for Biotechnology, University of Turku, Turku, Finland; 4 Department of Bacteriology and Immunology, Research Programs Unit, Immunobiology, University of Helsinki, and Helsinki University Hospital, Helsinki, Finland; 5 Biomaterials and Diagnostics Group, Turku University of Applied Sciences, Turku, Finland

**Keywords:** β2-microglobulin, Coxsackievirus A9, Receptor, HSPA5

## Abstract

**Background:**

Coxsackievirus A9 (CV-A9) is a pathogenic enterovirus type within the family *Picornaviridae*. CV-A9 infects A549 human epithelial lung carcinoma cells by attaching to the αVβ6 integrin receptor through a highly conserved Arg-Gly-Asp (RGD) motif, which is located at the exposed carboxy-terminus of the capsid protein VP1 detected in all studied clinical isolates. However, genetically-modified CV-A9 that lacks the RGD motif (CV-A9-RGDdel) has been shown to be infectious in some cell lines but not in A549, suggesting that RGD-mediated integrin binding is not always essential for efficient entry of CV-A9.

**Methods:**

Two cell lines, A549 and SW480, were used in the study. SW480 was the study object for the integrin-independent entry and A549 was used as the control for integrin-dependent entry. Receptor levels were quantitated by cell sorting and quantitative PCR. Antibody blocking assay and siRNA silencing of receptor-encoding genes were used to block virus infection. Peptide phage display library was used to identify peptide binders to CV-A9. Immunofluorescence and confocal microscopy were used to visualize the virus infection in the cells.

**Results:**

We investigated the receptor use and early stages of CV-A9 internalization to SW480 human epithelial colon adenocarcinoma cells. Contrary to A549 infection, we showed that both CV-A9 and CV-A9-RGDdel internalized into SW480 cells and that function-blocking anti-αV integrin antibodies had no effect on the binding and entry of CV-A9. Whereas siRNA silencing of β6 integrin subunit had no influence on virus infection in SW480, silencing of β2-microglobulin (β2M) inhibited the virus infection in both cell lines. By using a peptide phage display screening, the virus-binding peptide identical to the N-terminal sequence of HSPA5 protein was identified and shown to block the virus infection in both A549 and SW480 cell lines. HSPA5 was also found to co-localize with CV-A9 at the SW480 cell periphery during the early stages of infection by confocal microscopy.

**Conclusions:**

The data suggest that while αVβ6 integrin is essential for CV-A9 in A549 cell line, it is not required in SW480 cell line in which β2M and HSPA5 alone are sufficient for CV-A9 infection. This suggests that the choice of CV-A9 receptor(s) is dependent on the tissue/cellular environment.

**Electronic supplementary material:**

The online version of this article (doi:10.1186/s12985-016-0619-y) contains supplementary material, which is available to authorized users.

## Background

Enteroviruses are common human viruses and endemic in many developing countries. Although most enteroviral infections are subclinical, they may cause a wide spectrum of diseases including mild upper respiratory illness (common cold), febrile rash (hand, foot, and mouth disease and herpangina), aseptic meningitis, pleurodynia, encephalitis, acute flaccid paralysis (paralytic poliomyelitis) and neonatal sepsis-like disease [1, 2]. Coxsackievirus A9 (CV-A9) is an important member of *Enterovirus B* species (genus *Enterovirus B*, family *Picornaviridae*, http://www.picornastudygroup.com/) and one of the most prevalent and pathogenic enteroviruses [1, 3]. Structurally CV-A9 is a small, non-enveloped virus with a single-stranded RNA genome of 7452 nucleotides. The virus particle is composed of four structural proteins (VP1-4). VP1, VP2 and VP3 are located on the surface of the viral capsid, whereas VP4 faces the internal surface [4, 5]. A specific feature of the CV-A9 VP1 capsid protein is the C-terminal Arg-Gly-Asp (RGD) tripeptide motif through which the virus interacts with αV integrins *in vitro* and on the cell surface [4, 6].

Interaction with the cell surface is the primary event in the infectious viral entry. More than ten protein receptors are known to mediate the enterovirus entry, and it is generally believed that the binding of an enterovirus to the cell surface is mediated by the associations of one or more protein receptors and/or non-protein attachment factors [7, 8]. Successful cellular infection is thus dependent on both the attachment factors that may act as “sticky surface” and true protein receptors that contribute to the binding of a virus to the cell surface and intracellular transport of virus particles [9]. Many enteroviruses are capable of using several receptors for the cell binding, and/or cellular entry [10–12] but with the exception of Coyne and Bergelson study, it is not really known how different receptors interact during the internalization process.

RGD-binding integrins include five members of αV integrins (αVβ1, αVβ3, αVβ5, αVβ6 and αVβ8), two β1 integrins (α5β1 and α8β1) and the integrin αIIbβ3, and share the ability to recognize ligands, which contain the RGD tripeptide motif. There are four enterovirus types that possess an RGD motif in the VP1 protein [12] of which CV-A9 has been shown to bind *in vitro* to αVβ3 and αVβ6 integrins [13, 14]. Besides integrins there are other cell surface molecules that have been suggested to play a role in the CV-A9 infection. For example, β2-microglobulin (β2M, CD59), a major histocompatibility complex (MHC) class I heavy chain associated protein, and heat shock 70 kDa protein 5 (HSPA5 protein, also known as BiP or glucose-regulated protein 78 kDa, GRP78) have been shown to mediate the entry of CV-A9 [15–17]. Earlier, fluorescence resonance energy transfer (FRET) analysis suggested that the αVβ3 integrin and HSPA5 colocalize on the surface of green monkey kidney (GMK) cell line. This led to a hypothesis in which these receptors function in the binding of CV-A9 while β2M plays a role in the internalization step [16–18]. More recently, we have shown that CV-A9 possesses a high affinity only to the αVβ6 integrin and, therefore, have suggested it to be the primary binding/attachment receptor for the virus in A549 human epithelial lung carcinoma cell line [13]. The structural and functional features of the binding of αVβ6 integrin to CV-A9 have recently been demonstrated *in vitro* implying that the αVβ6 integrin acts as the binding receptor for the virus and that the virus binding to its integrin receptor does not induce uncoating and, further, viral RNA release [19]. Thus, there must be other molecules that mediate CV-A9 internalization and entry.

In this study, we used the human epithelial colon adenocarcinoma cell line (SW480) to analyze the cellular binding and the infectious entry of CV-A9. We provide evidence that β2M and HSPA5 are important in CV-A9 entry independently of the RGD-motif and αV integrins.

## Methods

### Cells and viruses

Human epithelial lung carcinoma (A549) cell line was obtained from American Type Culture Collection (ATCC). Human colorectal adenocarcinoma cells (SW480) [20] were from Dr. Stephen Nishimura (UCSF, USA). A549 and SW480 cells were maintained in DMEM and Ham’s F12 media, respectively, supplemented with 10 % foetal calf serum (FCS) (or 1 % for virus infections) and gentamycin. Coxsackievirus A9 (CV-A9, Griggs strain) [4, 21] and CV-A9-RGD-mutant (CV-A9-RGDdel) [22] were from laboratory collections. Viruses were propagated in A549 cells and purified as described previously [13, 23].

### Antibodies and proteins

CV-A9 antibodies were from laboratory collections [24, 25]. The function-blocking antibodies were against integrin αV (L230; ATCC), integrin αVβ3 (MAB1976Z; Chemicon®), integrin αVβ5 (MAB1961Z; Chemicon®), integrin αVβ6 (MAB2077Z; Chemicon®), integrin β1 (MAB2253; Chemicon®) and integrin α5β1 (MAB1969; Chemicon®). Antibodies to β2-microglobulin were from Santa Cruz Biotechnology (sc-51509). The rabbit antibody to HSPA5 protein (sc-13968) was from Santa Cruz. Alexa Fluor (AF) 488-, 546-, and the 568-labelled anti-mouse and anti-rabbit secondary antibodies were from Molecular probes. The horseradish peroxidase (HRP)-labelled anti-rabbit secondary antibody was from Pierce. In all immunofluorescence experiments, the nuclei were stained with Hoechst 33342 (Sigma-Aldrich). Purified integrin αVβ3 was obtained from BioMarket Ltd. (catalog item 01-INT-4). Integrin α5β1 was obtained from Chemicon® (catalog item CC1052). Integrin αVβ6 was produced and purified in Chinese hamster ovary (CHO) cells as described previously [26].

### Flow cytometry

The expression of integrin αVβ6, αVβ3 and β1 on the SW480 cell surface was analyzed by flow cytometry using specific monoclonal antibodies as previously described [13].

### Quantitation of integrin expression in A549 and SW480 cell lines

Total mRNA levels of integrin subunits β3, β6, and β1 were analyzed by quantitative reverse transcription-PCR (RT-qPCR) as previously described [27].

### Antibody blocking and *in vitro* binding assays

The methods have previously been described [13, 27]. In short, confluent cell monolayers (SW480 or A549 cells) were washed with a serum free cell medium before 1.5 μg of function-blocking αV-, αVβ5- and β1-integrin antibodies or anti β2-microglubulin (β2M) were added (dilutions were made in a serum free cell medium containing 1 mM MgCl_2_). After incubation in RT for 1 h, unbound antibodies were removed by washing and cells were incubated on ice for 1 h after the addition of the diluted virus. The virus dilution was set to achieve 10 % efficiency of infection in untreated cells. Unbound viruses were removed and infection was continued for 6 h before fixation (4 % formalin in PBS), permeabilization (0.1 % Triton-X100 in PBS) and staining (dilutions made in 3 % BSA in PBS) with virus-specific and secondary AF 488-labeled antibodies. Nuclei were stained with Hoechst and the ratio of the virus-specific signal to the total cell number was determined with Victor^3^ multilabel counter (Wallac, Finland). The cells infected in the absence of blocking antibodies were used as the positive infectivity control (100 % infection), and the mean was calculated from six parallel samples from three independent experiments. The maximal blocking effect was determined by using different concentrations (0–4 μg) of αV-integrin blocking antibodies in A549 cells where the CV-A9 infection depends on the integrin binding. The same experiment was performed with SW480 cells. In all data, error bars indicate standard deviation counted from 3 to 6 samples from three independent experiments and Anova single factor analysis was used to calculate the statistics.

In the *in vitro* binding method a solid-phase integrin binding assay was employed where the 96-well plate (Costar High Binding) was first coated with 300 ng integrins or BSA (a negative binding control). Wells were then blocked with 3 % BSA for 1 h. Following the addition of 0–200 ng of viruses, the plate was incubated at RT for 30 min and washed with the coating buffer after which the virus-specific antibody was added. After a 1 h incubation at RT, the wells were washed and the secondary anti-rabbit horseradish peroxidase conjugate (HRP; Pierce) was added. The wells were incubated for 45 min at RT, stained with H_2_O_2_, and the absorbance was read at 450 nm using Victor3. The mean was calculated from two parallel samples, range was calculated and the experiment was repeated twice with similar results.

### Receptor siRNA silencing and cell viability assay

In the receptor siRNA silencing assays, two individual siRNA molecules (Qiagen) for each gene were used (Additional file 1: Table S1). To transfect SW480 cells in 96-well plates, 1 pmol siRNA in 25 μl H_2_O was mixed with 0.4 μl siLentFect (Bio-Rad), diluted in a serum free medium and the wells were incubated at room temperature for 30 min. After incubation, 25,000 SW480 cells were added in the cell medium supplemented with 7 % serum and cultured for two days at 37 °C in 5 % CO_2_. The transfection conditions were optimized by transfecting the cells with siRNA targeting glyceraldehyde-3-phosphate dehydrogenase (GAPDH) and measuring the GAPDH enzyme activity with a KDalert GAPDH Assay Kit (Applied Biosystems). The virus inoculations aiming at 10 % efficiency of infection in untreated cells and antibody staining steps were performed as described above. The cell viability assay was performed according to manufacturer’s instructions as previously described [27]. Cells without CV-A9 served as a negative control and positive controls include non-transfected, mock-transfected and scramble-transfected cells.

### Phage display screening

Phage display screening with a peptide library displaying CX8C decapeptides (where X is any amino acid) was performed as previously described [28, 29]. Briefly, purified CV-A9 (50 μg ml^−1^ in PBS containing 0.5 mM MgCl_2_) was used to coat the wells on a Nunc Maxisorp 96-well plate. Specifically bound phages were eluted from the wells and used to infect K91kan *Escherichia coli*. Two consecutive rounds of panning were performed in the same manner. Individual cell clones were subjected to colony sequencing in which cell mass was directly used as template for PCR. During the denaturation step, cells are lysed releasing the nucleic acid for amplification. Primers 5′ and 3′ to the peptide insertion site of the phage were used: the forward primer was 5′-TAA TAC GAC TCA CTA TAG GGC AAG CTG ATT AAC CGA TAC AAT-3′ and the reverse primer 5′-CCC TCA TAG TTA GCG TAA CGA TCT-3′. Sequences were compared against SwissProt database using the FASTA program.

### Peptide blocking assay

CV-A9 was mixed with synthetic ESPLSLVA (20 μM 200 μM and 5 mM) or RRRGEL peptides (5 mM) and incubated for 30 min. Both peptides and virus were diluted into PBS with 1 mM MgCl_2_. The RRRGEL-peptide was used as a negative control because it does not block CV-A9 infection [30]. After incubation, CV-A9 was added onto the cells in the presence of peptides and incubation was continued for another 30 min on ice. Instead of using MOI value, the virus amount was set to achieve 10 % efficiency of infection in untreated cells. Unbound viruses were removed, a pre-warmed infection medium was added and the plate was transferred into 37 °C for 6 h. The cells were then fixed, permeabilized and stained with the virus specific antibody and secondary AF 488-antibody. Nuclei were stained with Hoechst and the virus and Hoechst signals were measured with Victor3 multilabel counter. The cells incubated without peptide were used as a positive infection control and error bars indicate standard deviation counted from four independent samples. Anova single factor analysis was used to calculate the statistic and *P* < 0.05 was considered as a significant result. The experiment was repeated twice with a similar outcome.

### Confocal imaging

In all endocytosis assays, SW480 cells were grown on cover slips in the wells of 24 well plate overnight at 37 °C after which the plate was transferred onto ice and cells were infected with the virus aiming at 40-60 % efficiency of infection. The infection was followed at 37 °C from 0 min up to 6 h, after which the cells were fixed and permeabilized (excluding the 0-min time period). Antibody staining was conducted at room temperature by using specific antibodies diluted in PBS with 3 % BSA. The plate was incubated at RT for 1 h in the presence of primary antibodies after which the cells were washed and AF-conjugated secondary antibodies were added. After 30 min incubation at RT, the cells were washed and nuclei stained with Hoechst 33342. Fixed, permeabilized and immunostained cells were then mounted on microscope slides in Mowiol 4–88 (Calbiochem-Novabiochem), 25 % glycerol, 0.1 M Tris-HCl, pH 8.5, containing Dabco 25 mg ml^−1^ (Sigma-Aldrich) and examined with Zeiss LSM510 META confocal microscopes using a Plan-Apochromat objective (63x oil). Colocalization analyses (automatic thresholding after background subtraction, Costes P-value calculation with 100 iterations) of selected image stacks were performed with BioImageXD software [31]. Scale bars are shown in the figures.

## Results

### CV-A9 and genetically modified RGD-deficient CV-A9 infect SW480 cells

**Fig 1:**
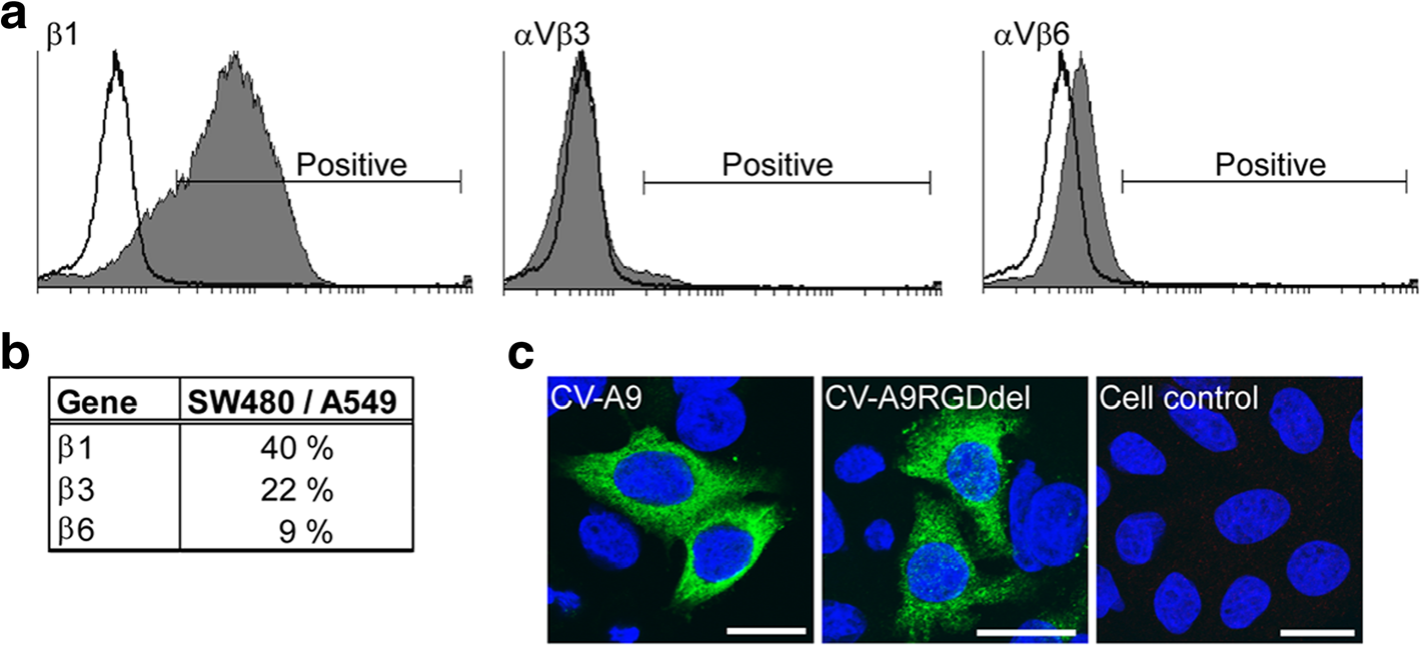
CV-A9 infection in the SW480 cell line is independent of an RGD and αV-integrins. **a** Fluorescence from flow cytometric analysis of integrin expression. SW480 cells were incubated with integrin specific monoclonal antibodies against β1, αVβ3 or αVβ6 (cell controls as shown in a white plot were incubated with a secondary antibody) and 10,000 cells were measured in each sample. The data was analyzed by the Cyflogic program and the area of positive signal is indicated in a white plot. In the case of αVβ3 the white area is shadowed by the negative dark background. Standard threshold values were used in the assays. **b** The ratio (SW480/A549) of integrin mRNA levels measured by quantitative PCR is indicated. **c** Immunofluorescence images of SW480 cells infected with the wild type CV-A9 and with the RGD-deletion mutant (CV-A9RGDdel). Antibodies specific to CV-A9 (*green*) were used. The nuclei (*blue*) were stained with Hoechst. The scale bar = 20 μm

Previously, it has been shown that infection of CV-A9 into A549 cells is dependent on integrin αVβ6 [27]. To study CV-A9 infection in a cellular model in which αVβ6 is not expressed, we chose to use human colon adenocarcinoma cells (SW480) [20]. It was reported by plaque titration assay that CV-A9 infects SW480 cells only after transfection with the cDNA copy of the β6 integrin subunit and overexpression of the corresponding protein [14]. However, the recent paper by Gianni et al. [32] shows that there are several lines of SW480 cells, some of which express αVβ6 and some which do not. Therefore, the integrin expression profile of SW480 cells used in this study was analyzed by flow cytometry using antibodies specific to integrin β1-, αVβ3- and αVβ6 (Fig. 1a). Integrin β1 subunits were highly expressed whereas αVβ3 and αVβ6 integrin were not detected or were expressed to a much lesser extent in the SW480 cells than in the A549 cells [13]. This is in agreement with the previous reports where the SW480 cell line was used [14, 33, 34]. The mRNA levels of β1- (used as a positive control), β3- and β6-subunits were also measured by quantitative PCR (qPCR) and the results were compared with the mRNA levels in the A549 cell. The results are shown in Fig. 1b as a percentage of the mRNA levels in SW480 to A549 cells [27]. Corresponding mRNAs of β3 and β6 integrin subunits were present in the SW480 cells but to a much lesser extent than in the A549 cells (Fig. 1b). The expression level of β6-subunit in SW480 was only 9 % of the level detected in the A549 cells. The data indicate that αVβ3 and αVβ6 are only weakly expressed on the surface of SW480 cells, which is also in line with another study in which foot-and-mouth disease virus was studied in SW480 cell line [35]. To further demonstrate that CV-A9 infects these SW480 cells independently of an RGD-mediated integrin binding, we infected both the prototype CV-A9 (Griggs strain) and CV-A9-RGDdel, a virus mutant lacking the RGD motif [13, 22], into SW480 cells, and followed the infection by using cell imaging techniques. Figure 1c shows that CV-A9-RGDdel is capable of entering SW480 cells similarly to CV-A9.

### Anti-integrin antibodies do not block CV-A9 infection in SW480 cells

**Fig. 2:**
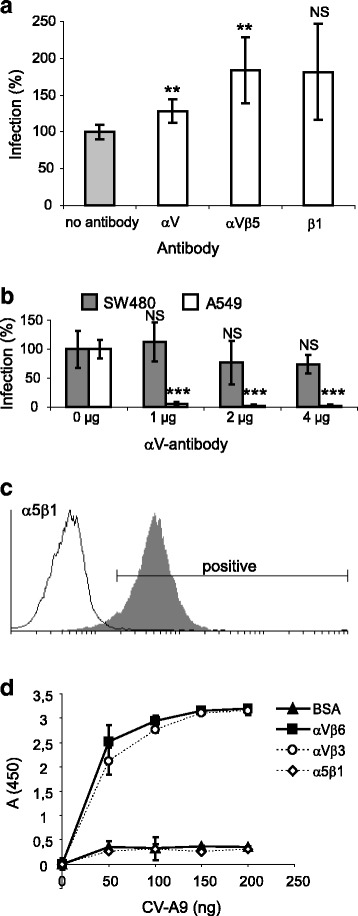
RGD-binding integrins are not required for the CV-A9 infection in the SW480 cells. **a** Blocking of the CV-A9 infection with antibodies against αV, αVβ5 or β1. The ratio of cells exhibiting a fluorescent signal specific for the virus signal to Hoechst-stained nuclei was determined by a Victor^3^ multilabel counter. Cells infected in the absence of integrin antibodies (panel on the left) were used as a positive infectivity control (infectivity set to 100 %). Error bars indicate standard deviation counted from three independent experiments with 6 parallel samples. Significance is indicated with an asterisk; *P < 0.05, **P < 0.01, ***P < 0.001. NS = not significant. **b** αV-antibody blocking in the SW480 and A549 cells. Error bars indicate standard deviation counted from 3 to 6 samples from three separate experiments and significant reduction is indicated with an asterisk; ***P < 0.001. **c** Fluorescence from cytometry analysis. SW480 cells were incubated with the integrin specific monoclonal antibody against α5β1 and secondary antibody and the cell control shown in a white plot was incubated only with a secondary antibody. The result was analyzed by the Cyflogic program. **d** The integrin binding assay with αVβ6, αVβ3, α5β1 or BSA (as a control). CV-A9 was stained with polyclonal primary and secondary antibodies. The range from the two parallel samples is shown

To exclude the possibility that low level of αV-integrin expression (undetectable by cell sorting analysis in Fig. 1a) in SW480 cells could explain the reduced CV-A9 infection, the integrin-independent infectivity of CV-A9 was further analyzed by using anti-integrin blocking antibodies (Fig. 2). As expected, anti-αV antibodies significantly (*P* = 1.8–2.3 × 10^−5^) blocked the CV-A9 infection in the A549 but not in the SW480 cells (*P* = 0.2–0.6), while antibody blocking of integrin αVβ5 and β1 did not reduce the infectivity (Fig. 2a-b). In addition, α5β1 did not bind to the virus in *in vitro* assay although it was highly expressed in SW480 cells (Fig. 2c-d). These data together with CV-A9/CV-A9-RGDdel infectivity results suggest that the CV-A9 entry into SW480 cells occurs in the absence of αV integrins.

### Receptor siRNA assay and antibody blocking of β2-microglobulin

**Fig 3:**
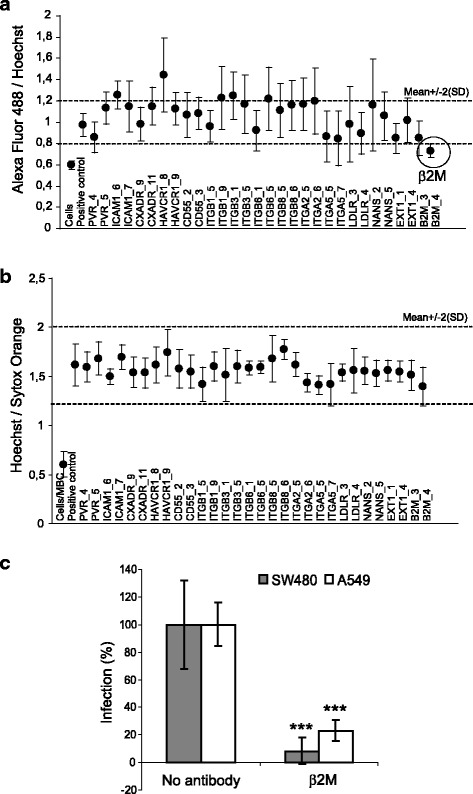
β2-microglobulin (β2M) is essential for the CV-A9 infection. **a** The siRNA-transfected SW480 cells were cultured for 48 h and then infected with CV-A9. The infection was followed for 6 h and visualized by CV-A9-specific antibodies. The ratio of the AF 488 signal to the Hoechst (nuclei) signal was considered as the measure of efficiency of the infection. The experiment was repeated five times with mean values calculated. The error bars indicate standard deviation and the cut-off values were calculated as positive control mean ± 2 × SD. Positive control includes non-transfected, mock-transfected and scramble-transfected cells. **b** The cell viability was checked by staining the cells with Hoechst and Sytox Orange. The cells treated with 10 mM methyl-β-cyclodextrin (MBC) were used as “cell death” cytotoxicity controls. **c** SW480 and A549 cells were incubated in the presence of a function blocking β2M antibody. The infection % was calculated as a ratio of the virus signal to the Hoechst signal. The cells infected with CV-A9 in the absence of the antibody were used as positive infection controls (100 % infection). Error bars indicate the standard deviation from three independent experiments with 4–6 parallel samples and the significance reduction in infectivity is indicated with an asterisk. (*P* = 5.6 × 10^−5^ in SW480 cells and *P* = 2.1 × 10^−5^ in A549 cells)

An siRNA panel against known picornavirus receptor proteins was developed to identify receptors that would mediate CV-A9 infection in SW480 cells [27] (Additional file 1: Table S1). Two individual siRNA molecules for each target gene were used. As shown in Fig. 3a, the most effective inhibition of the CV-A9 infection in the SW480 cells was obtained by using the siRNA targeted against β2-microglobulin (β2M), which is in line with the previous results obtained using the A549 cells [27]. As expected, none of the RGD-binding integrins had an effect on the CV-A9 infectivity in the SW480 cell line while in the A549 cells the silencing of the αVβ6 integrin blocked the CV-A9 proliferation [27]. To evaluate the viability of the cells after the siRNA transfections, the cells were stained with the dead-cell indicator Sytox Orange. Methyl-β-cyclodextrin (MBC) was used as a cell death marker because at 10 mM concentration this inhibitor is lethal to the cells. None of the used siRNAs showed cytotoxicity indicating that the observed changes in the virus proliferation were due to the silencing of a specific gene, and not due to the overall effects on the cell viability (Fig. 3b).

The role of β2M in CV-A9 entry into SW480 cells was further analyzed by using β2M antibodies. As shown in Fig. 3c, β2M-antibodies had a clear inhibitory effect on the CV-A9 infection in both the SW480 and A549 control cells. Antibodies significantly reduced the CV-A9 infectivity down to 10 % (*P* = 5.6 × 10^−5^) in the SW480 and 20 % (*P* = 2.1 × 10^−5^) in the A549 cell lines. Furthermore, the reduction in infectivity occurred in a dose-dependent manner (Additional file 2: Figure S1). Thus, the data suggest that β2M is essential for a successful CV-A9 infection in both SW480 and A549 cell lines.

### Role of HSPA5 in CV-A9 infection

**Fig 4:**
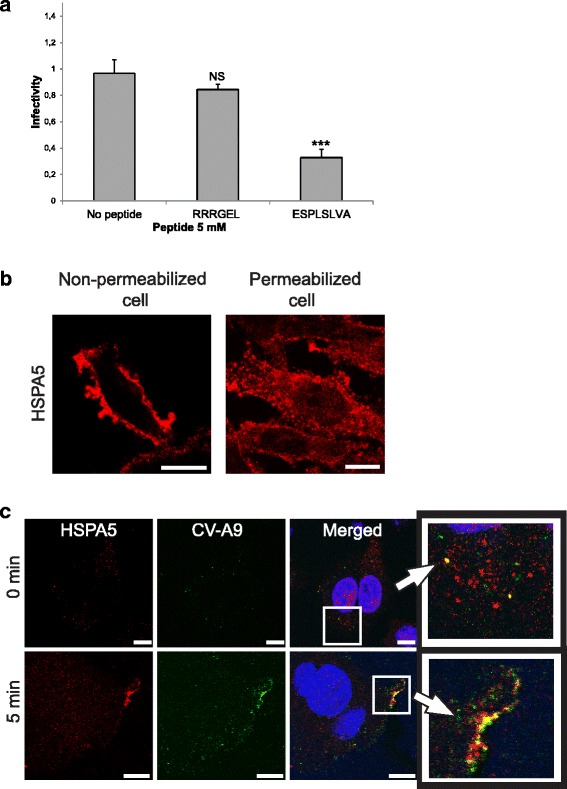
Co-endocytosis of HSPA5 with CV-A9. **a** The peptide blocking assay. The infectivity of CV-A9 was blocked by the ESPLSLVA peptide and RRRGEL was used as negative control. Cells were fixed six hours post-infection, permeabilized and the virus was stained with specific antibodies. Nuclei were stained with Hoechst and the infectivity was measured with Victor^3^ multilabel counter. Error bars indicate the standard deviation from four independent samples and significance *P* = 4.0 × 10^−5^ is indicated with asterisk (***) or stated as not significance (NS). **b** Confocal imaging of abundance of HSPA5 on the surface of SW480 cells (non-permeabilized cell) and in the cell interior (permeabilized cell). Non-infected cells were stained with the HSPA5 specific antibody shown in red. **c** Co-internalization of CV-A9 and HSPA5. The cells were stained with virus-specific (*green*) and HSPA5-specific (*red*) antibodies in non-permeabilized (0 min) cells to show the cell surface binding or in permeabilized cells (5 min) to show the CV-A9/HSPA5 internalization. The nuclei were stained with Hoechst (*blue*). Co-localization of CV-A9 and HSPA5 is visualized by *yellow color* in the merged images. Scale bars are 10 μm

In addition to siRNA assay, a peptide phage display screening was employed to identify peptides that bind to the virus particles. The potential peptide binders of CV-A9 were selected from the library of cyclic, degenerate CX8C peptides displayed on a filamentous phage particle [28], and ten CV-A9 and CV-A9RGDdel-binding phages were sequenced. Only a single peptide binder, ESPLSLVA, proved to be a potential viral receptor candidate for CV-A9, while the sequences of the other peptides did not align with any known cellular virus receptors and were not analyzed further (Table 1). Interestingly, ESPLSLVA resides in the N-terminal sequence of the HSPA5 protein (NM_005347.4) [36]. Previous studies have demonstrated that the HSPA5 protein and CV-A9 co-immuno-precipitate in cell lysates from green monkey kidney (GMK) cells and antibodies against the protein block the CV-A9 infection [17], suggesting a physical interaction between HSPA5 and the virus. The ability of ESPLSLVA peptide to prevent the virus infection was hence tested. In the peptide blocking assay where high quantities of ESPLSLVA peptide was used the CV-A9 infection in the SW480 cells was significantly (*P* = 4.0 × 10^−5^, respectively) blocked by the peptide while the control peptide did not affect infectivity (Fig. 4a). Interestingly, HSPA5 peptide blocked CV-A9 infection in both SW480 and A549 cell lines in a dose-dependent manner, which suggests that HSPA5 possesses a general role in CV-A9 infection (Additional file 3: Figure S2).

**Table 1:** Peptide phage display panning using CV-A9 and CV-A9-RGDdel as targets

CV-A9	CV-A9-RGDdel	Non-specific
ESPLSLVA (1)	LSWWSRKW (1)	NWWSPVGV (1)
WWGIWMQE (1)	WWAIWMQE (1)	GWFKWGLW (1)
NLWGFWFP (2)	LWWQIWDG (4)	RPWPFWWQ (2)
PWWWGRNV (6)	PWWWGRNV (3)	FLGFPHWV (1)
	WIWAWRSS (1)	LGRWWWWS (2)
		GWLWPGWF (1)
		LQFSFLGF (2)

To further study the role of HSPA5 in CV-A9 infection, we performed experiments by using immunofluorescence confocal microscopy to localize CV-A9 and HSPA5 during the early stages of the virus infection. HSPA5 protein was visualized by antibody staining in the absence of the virus in both non-permeabilised and permeabilised cells to demonstrate that it was expressed on the surface of the SW480 cells (Fig. 4b). The distribution of HSPA5 was drastically different between the infected and non-infected cells (Fig. 4b-c). The HSPA5 protein accumulated in visible spots (at 0 min), which partially co-localized (29 %) with CV-A9. This indicates early interaction of the virus with the receptor on cell surface. Moreover, after 5 min post-infection, a clear co-localization (40 %) between the HSPA5 and CV-A9 was detected at close vicinity to the inner surface of plasma membrane, further suggesting that HSPA5 has a role in the CV-A9 attachment to the cell surface and in the early stage of the internalization. In conclusion, the data suggest that HSPA5 acts both in the attachment and entry stages of CV-A9 in the SW480 cells.

## Discussion

In this paper we used human colon adenocarcinoma cell line and demonstrate that both the native CV-A9 and its RGD-deletion mutant (CV-A9RGDdel) internalize into SW480 cells (Fig. 1). In addition, anti-integrin function-blocking antibodies were not capable of blocking the CV-A9 infection of SW480 cells (Fig. 2). Thus, it is likely that CV-A9 infection of the SW480 cells is independent of the RGD-binding αVβ3 and αVβ6 integrins, which have previously been suggested to play a role in CV-A9 infection in other cell lines. These data raised the question as to which cellular receptors mediate CV-A9 infection in SW480.

CV-A9 possesses an RGD motif in the C-terminus of the VP1 capsid protein through which the virus has been shown to bind αVβ3 and/or αVβ6 integrins *in vitro* [13, 14, 22]. However, it has also been demonstrated that the virus is capable of entering cells in an RGD-independent manner, i.e. removal of the RGD motif does not interfere with virus infection in certain cell lines [6, 22]. However, the mechanism or mediators have remained unknown. CV-A9 infection is also dependent on attachment factors or co-receptors. An infection model using green monkey kidney cell line has been proposed [17] by which CV-A9 utilizes the αVβ3 integrin as its primary attachment receptor and HSPA5 as its co-receptor. According to this model, αVβ3 integrin and HSPA5 mediate CV-A9 attachment while HSPA5 cross-links MHC I concentrating the molecules at the same location on the cell surface. In contrast, we have recently suggested that integrin αVβ6 acts as a high affinity receptor for CV-A9 in A549 cells [13, 27], and it may thus function solely as a binding receptor. We also demonstrated that β2M confines the virus to the peripheral regions of the A549 cells [27], which was also the case in SW480 cells (this study). This suggests that β2M has a role either in the attachment or in the early stage of the internalization regardless of integrins.

The results from the phage display screening and peptide blocking assay propose that CV-A9 also interacts with the HSPA5 protein on the SW480 cell surface, which thus seems to serve as a true protein receptor for the virus (Fig. 4). While HSPA5 resides primarily in the endoplasmic reticulum [37], it is also expressed on the surface of various cell types [17, 38, 39]. Indeed, *Borna disease virus* (BDV) interacts with surface-exposed HSPA5 through the GP1 capsid protein [40], and antibodies directed against the N-terminus of HSPA5 protein have also been shown to inhibit the binding and infection of the dengue virus serotype 2 [41]. Interestingly, HSPA5 seems also to be effective in A549 cell line (Additional file 3: Figure S2) where integrin αVβ6 is required for infectivity [27]. Given that HSPA5 possesses a role in the entry stage of different viruses, it may be a common factor in cellular endocytosis of many viruses.

So far, most cellular studies related to CV-A9 have been carried out in cancerous cell models in which the receptor expression may differ from native cells. It is hence possible that when infecting humans, CV-A9 uses different receptors in a tissue-specific manner, which allows the virus to survive in a multicellular environment. Because many enteroviruses use several receptors for the cell binding, and/or cellular entry [10–12], it is tempting to speculate what happens in clinical CV-A9 infection. Since the RGD motif is conserved in clinical CV-A9 isolates [42] it is likely that RGD-αV-integrin interaction plays a significant role in initiating CV-A9 life cycle in multicellular environment. However, the role of integrin may be solely in binding the virus to the cell surface. We have previously suggested that integrin αVβ6 is high affinity binding receptor to CV-A9 [13, 19], but it seems that virus does not uncoat upon integrin binding [19] and it is not internalized with the integrin receptor [27]. Thus, other receptors are needed for the internalization process. It is possible that when in contact with epithelial cells, CV-A9 binds to αVβ6 integrin via its RGD motif that concentrates the virus on the cell surface, and interaction with the HSPA5 and β2M proteins further mediate the virus internalization. HSPA5 and β2M may play central roles in CV-A9 infection because they are ubiquitously expressed in most cell types [43].

## Conclusions

We conclude that CV-A9 infection in SW480 cells is not dependent on αV integrins, but requires β2M and HSPA5. The data suggest that CV-A9 may use alternate receptor(s) in different cellular environment, and may partially explain wide tropism and pathogenicity of this enterovirus type. The data also suggest that β2M and/or HSPA5 may possess a general role as a viral receptor.

## Additional files

Additional file 1: Table S1. The list of siRNA used in the study. (PDF 209 kb)
Additional file 2: Figure S1. CV-A9 infection is blocked dose-dependently by β2M antibody in SW480 and A549 cell lines. Significance reduction in virus infectivity is shown with an asterisk (** < 0.01; *** < 0.001) and error bars indicate standard deviation counted from four parallel samples. (PDF 61 kb)
Additional file 3: Figure S2. In A549 and SW480 cells, CV-A9 infection is significantly blocked by peptide in a dose-dependent manner. Significant reduction is shown with an asterisk (* < 0.05) and error bars indicate standard deviation counted from four parallel samples. (PDF 6 kb)

## Abbreviations

A549: Human epithelial lung carcinoma
ATCC: American type culture collection
CV-A9: Coxsackievirus A9
FCS: Foetal calf serum studies
FRET: Fluorescence resonance energy transfer
GMK: Green monkey kidney
HSPA5: Heat-shock 70kDa protein 5
MHC: Major histocompatibility complex
qPCR: Quantitative polymerase chain reaction
RD: Rhabdomyosarcoma
SW480: Human epithelial colon carcinoma
β2M: β2-microglobulin
