# Supplementary material for: Integrins are not essential for entry of coxsackievirus A9 into SW480 human colon adenocarcinoma cells
Source: Virol J. 2016 Oct 18;13:171. doi: 10.1186/s12985-016-0619-y (PMC5069866; doi:10.1186/s12985-016-0619-y)
Supplement: Additional file 1: Table S1. — The list of siRNA used in the study. (PDF 209 kb) [file 12985_2016_619_MOESM1_ESM.pdf]

Additional file 1 The list of siRNAs used in the study

Gene	NCBI GeneID	Protein, aliases	Viruses	siRNA
		Negative control siRNA		AllStars 0001027280
PVR	5817	CD155, PVR	Polio 1,2,3	Hs_PVR_4<br>SI00089110<br><br>Hs_PVR_5<br>SI03070809
ICAM1	3383	ICAM-1, Intracellular adhesion molecule-1, CD54	CV-A13, -18, -21<br><br>Major group rhinoviruses	Hs_ICAM1_6<br>SI03093923<br><br>Hs_ICAM1_7<br>SI03110576
CXADR	1525	CAR, Coxsackievirus-adenovirus receptor	CV-B1, CV-B2, CV-B3, CV-B4, CV-B5, CV-B6	Hs_CXADR_9<br>SI03023447<br><br>Hs_CXADR_11<br>SI03106495
HAVCR1	26762	HAVcr-1; HAV cellular receptor	HAV	Hs_HAVCR1_8<br>SI03093223<br><br>Hs_HAVCR1_9<br>SI03100713
CD55	1604	DAF, decay accelerating factor, CD55	E-3, -6, -7, -11, -12, -13,<br><br>-20, -21, -24, -29, -30<br><br>EV-70<br><br>CV-B1, CV-B3	Hs_CD55_2<br>SI03075660<br><br>Hs_DAF_3<br>SI00012103
ITGB1	3688	Integrin β1	E-1<br><br>FMDV<br><br>HPeV-1	Hs_ITGB1_5<br>SI00300573 (92%)<br><br>Hs_ITGB1_9<br>SI02662590 (92%)
ITGB3	3690	Integrin β3	CV-A9, FMDV, HPeV-1	Hs_ITGB3_1<br>SI00004585<br><br>Hs_ITGB3_5<br>SI02623159
ITGB6	3694	Integrin β6	CV-A9, FMDV	Hs_ITGB6_1<br>SI00017801<br><br>Hs_ITGB6_5<br>SI03053029

ITGB8	3696	Integrin β8	FMDV	Hs_ITGB8_5<br>SI03030174<br><br>Hs_ITGB8_6<br>SI03066623
ITGA2	3673	Integrin α2	E-1	Hs_ITGA2_5<br>SI02664081 (86%)<br><br>Hs_ITGA2_6<br><br>SI02664088 (87%)
ITGA5	3678	Integrin α5	FMDV	Hs_ITGA5_5<br>SI02654841 (86%)<br><br>Hs_ITGA5_7<br>SI03071572
LDLR	3949	LDL-R, Low-density lipoprotein receptor	Minor group rhinoviruses	Hs_LDLR_3<br>SI00011179<br><br>Hs_LDLR_4<br>SI00011186
NANS	54187	N-acetylneuraminic acid synthase (sialic acid synthase), SAS	HRV-87 (sialic acid)	Hs_NANS_2<br>SI00654850<br><br>Hs_NANS_5<br>SI04146345
EXT1	2131	exostoses (multiple) 1;<br><br>(an ER-resident type II transmembrane glycosyl-transferase involved in the chain elongation step of heparan sulfate biosynthesis)	FDMV	Hs_EXT1_1<br>SI00002562<br><br>Hs_EXT1_4<br>SI00002583
B2M	567	Beta-2-microglobulin	CV-A9, E-1	Hs_B2M_3<br>SI00059038<br><br>Hs_B2M_4<br>SI00059045
