# Supplementary figures and images for: Integrins are not essential for entry of coxsackievirus A9 into SW480 human colon adenocarcinoma cells
Source: Virol J. 2016 Oct 18;13:171. doi: 10.1186/s12985-016-0619-y (PMC5069866; doi:10.1186/s12985-016-0619-y)

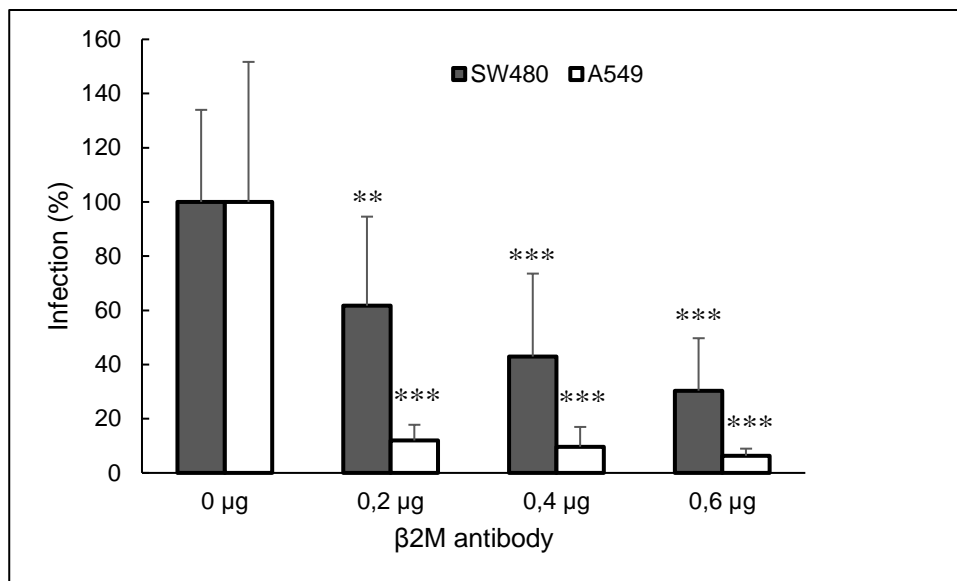

Supplement: Additional file 2: Figure S1. — CV-A9 infection is blocked dose-dependently by β2M antibody in SW480 and A549 cell lines. Significance reduction in virus infectivity is shown with an asterisk (** < 0.01; *** < 0.001) and error bars indicate standard deviation counted from four parallel samples. (PDF 61 kb) [file 12985_2016_619_MOESM2_ESM.pdf]

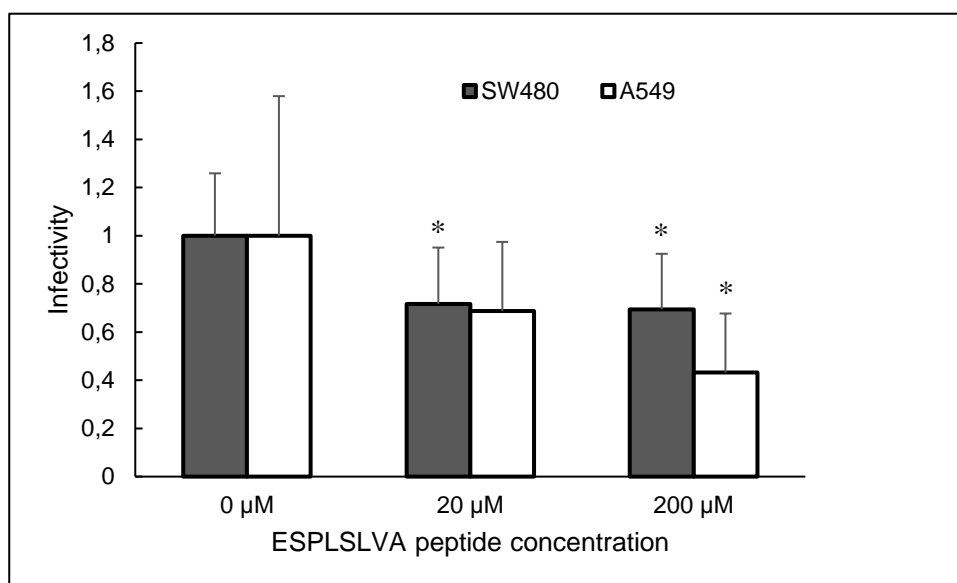

Supplement: Additional file 3: Figure S2. — In A549 and SW480 cells, CV-A9 infection is significantly blocked by peptide in a dose-dependent manner. Significant reduction is shown with an asterisk (* < 0.05) and error bars indicate standard deviation counted from four parallel samples. (PDF 6 kb) [file 12985_2016_619_MOESM3_ESM.pdf]
